# Violence in the Family of Origin, Reflective Functioning, and the Perpetration of Isolating Behaviors in Intimate Relationships: A Mediation Model

**DOI:** 10.3390/bs15030288

**Published:** 2025-02-28

**Authors:** Tommaso Trombetta, Maria Noemi Paradiso, Fabrizio Santoniccolo, Luca Rollè

**Affiliations:** Department of Psychology, University of Turin, 10124 Turin, Italy; tommaso.trombetta@unito.it (T.T.); fabrizio.santoniccolo@unito.it (F.S.); l.rolle@unito.it (L.R.)

**Keywords:** intimate partner violence, isolating behaviors, perpetration, violence in the family of origin, mentalization, reflective functioning

## Abstract

Background: The intergenerational transmission of violence from the family of origin to couple relationships in adulthood is well-known in the scientific literature. However, the perpetration of isolating behaviors (IBs) is still poorly explored, and additional studies are required to comprehend the mechanisms that intervene in the association between Violence in the family of origin (VFO) and isolating behaviors. Drawing from Fonagy’s mentalization model, which describes reflective functioning as the capacity to conceive mental states as explanations for one’s own and other people’s behavior, the present study aims to examine the mediating role of reflective functioning in the link between VFO and the perpetration of isolating behaviors. Methods: Online self-report questionnaires were completed by 663 Italian participants (66.8% women; *M*_age_ = 28.4, *SD* = 8.5) who were in a couple relationship in the last 12 months. A mediation model was tested to assess the direct and indirect effects of VFO on the perpetration of isolating behaviors through certainty and uncertainty of mentalization. Results: A direct association was found between VFO and the perpetration of isolating behaviors. Furthermore, we found an indirect effect of VFO on the perpetration of the perpetration of isolating behaviors, mediated by the certainty and uncertainty of mentalization. Conclusions: The results demonstrated the mediating role of reflective functioning in the intergenerational transmission of violence and support its implication in the perpetration of isolating behaviors in couple relationships. Although these results are preliminary, they can provide useful data at the theoretical and clinical levels.

## 1. Introduction

Intimate partner violence (IPV) is a significant public health problem that frequently results in adverse physical and psychological consequences for the victims (Plichta, 2004; Black, 2011; World Health Organization, 2021). Following the World Health Organization (2021) definition, IPV includes any behaviors that induce physical, sexual, or psychological harm. Specifically, these behaviors include physical abuse, sexual coercion, psychological abuse, and controlling behaviors. Compared to physical, sexual, and psychological IPV, little is known about controlling behaviors. While psychological violence includes acts such as insults, humiliation, intimidation, and threats, controlling behaviors encompass a range of behaviors intended to make a victim dependent on the perpetrator, such as depriving them of financial resources, and displaying extreme jealousy and possessiveness (Walker et al., 2021; World Health Organization, 2021). Furthermore, controlling behaviors also include isolating behaviors intended to exclude the victim from their social network (Walker et al., 2021).

Suffering controlling behaviors can have serious consequences. Notably, psychological effects on victims, such as fear, anxiety, low self-esteem, depression, and post-traumatic stress disorder, are documented (Aizpurua et al., 2021; Kelly & Johnson, 2008). The experience of controlling behaviors is also associated with suicidal thoughts and poor relationships with the victim’s social network (Krantz & Vung, 2009; Ali et al., 2014). However, the scientific literature has only just begun to address the perpetration of controlling behaviors (Aizpurua et al., 2021; Solinas-Saunders, 2022; Walker et al., 2021), and specifically the perpetration of isolating behaviors (Trombetta et al., 2023). Aware of this gap in the scientific literature and considering the negative consequences related to controlling behaviors, the current study aims to investigate the perpetration of isolating behaviors that, despite being understudied, have already been shown to have a detrimental impact on victims’ dependence on the perpetrator (De Sousa et al., 2022). The data can provide clinically relevant insights to reduce the spread of controlling behaviors and limit recidivism.

Several studies (Kwong et al., 2003; Delson & Margolin, 2004; Eriksson & Mazerolle, 2014; Rivas-Rivero & Bonilla-Algovia, 2022) have found an association between exposure to violence in the family of origin (VFO) and the perpetration of IPV in adulthood, emphasizing the concept that violence may be passed down through the generations. According to attachment theory, parents exhibiting frightening, aloof, and unpredictable parenting methods make it more difficult for their infants to develop secure bonds (Juan et al., 2020). In addition, experiencing violence in the family of origin not only promotes insecure and disorganized attachment but also creates internal working models of families that incorporate violence (McClellan & Killeen, 2000). Since exposure to these behaviors increases their repeatability, children acquire them through imitation (Rivas-Rivero & Bonilla-Algovia, 2022). In line with Bandura’s (1973) social learning theory, people who grow up in abusive families are taught that using aggressiveness to resolve problems with others is acceptable. Consequently, people are more likely to engage in violent behaviors when they are exposed to violence because it supports a belief system that legitimates the use of violence in intimate relationships (Eriksson & Mazerolle, 2014). However, how early experiences of violence could turn into the potential for violence is still poorly understood. Specifically, the role of dimensions of psychological functioning (e.g., emotion regulation, reflective functioning) in the intergenerational transmission of violence from the family of origin to intimate relationships in adulthood deserves further investigation. In addition, while several studies (Eriksson & Mazerolle, 2014; Elmquist et al., 2016; Haj-Yahia et al., 2021) have investigated the relationship between violence in the family of origin and IPV perpetration, focusing specifically on physical and psychological violence, as far as we know, no studies to date have assessed the intergenerational transmission of violence specifically considering the perpetration of isolating behaviors.

Mentalization could be one of the mechanisms involved in the relationship between violence in the family of origin and the perpetration of isolating behaviors. Mentalization, operationalized as reflective functioning (RF), refers to “the mental process by which an individual implicitly and explicitly interprets the actions of himself and others as meaningful based on intentional mental states such as personal desires, needs, feelings, beliefs, and reasons” (Bateman & Fonagy, 2004, p. XXI). Traditionally, reflective functioning has been measured through the Reflective Functioning Scale (RFS; Fonagy et al., 1998) using narratives from the Adult Attachment Interview (George et al., 1985). However, in 2016, in order to collect more data and to increase the generalizability of the data, Fonagy and colleagues developed the Reflective Functioning Questionnaire (RFQ; Fonagy et al., 2016), a self-report instrument which evaluates two dimensions of mentalization: the certainty (RFQ_C) and uncertainty (RFQ_U) of mentalization. Low levels of certainty of mentalization correspond to maladaptive hypermentalizaing (i.e., excessive certainty of mentalization), while high levels refer to adaptive levels of certainty about mental states. On the contrary, low levels of uncertainty of mentalization correspond to the adaptive uncertainty abouth mental states resulting from their partial inscrutability, while high levels refer to maladaptive hypomentalizing (i.e., concrete thinking and lack of understanding of the mental states of self and others).

Fonagy’s mentalization model is deeply rooted in attachment theory. Indeed, a child’s ability to comprehend their intrapsychic experiences, to understand other people’s behaviors in terms of mental states, and consequently to regulate their emotions is predicted by the quality of their early attachment relationships (Fonagy, 1999; Luyten et al., 2017). During the early stages of infancy, since a child’s primary mode of communication is non-verbal and crying is a way for them to express their anguish, the caregiver’s emotion regulation is crucial (Schultheis et al., 2019). Initially, the caregiver’s capacity to co-regulate emotional states is essential for the development of a child’s reflective functioning skills and for their ability to regulate emotions autonomously (Schwarzer et al., 2021). Moreover, the caregiver’s sensitive interactions let the child see their emotional states as significant and predictable (Schwarzer et al., 2021). In secure attachment relationships, the caregiver can see their child as an intentional being, recognize their emotional states, and properly respond to their needs, enabling the infant to develop a coherent representation of their inner world (Parada-Fernández et al., 2021). A child can form representations of their own subjective experience when their caregiver appropriately reflects their subjective experience (i.e., how well attachment figures mirror the infant’s subjective experience, displaying affects in a contingent way) (Fonagy & Luyten, 2018). This experience of “themselves in the caregiver’s mind” (Fonagy, 1999) is crucial for the development of the infant’s sense of self (Luyten et al., 2020) and for emotion regulation capacities (Bateman & Fonagy, 2012). In contrast, insecure attachment relationships—which refer to the experience of not receiving sensitive responses to one’s attachment needs—can hinder the development of reflective functioning (Asen & Fonagy, 2021).

Conversely, as underlined by several studies (Asen & Fonagy, 2017a; Garon-Bissonnette et al., 2023; Stover et al., 2022), adverse childhood experiences, such as violence in the family of origin, may disrupt emotion regulation strategies and reflective functioning capacities. In these cases, caregivers may promote maladaptive emotion regulation strategies and harming behaviors as a coping mechanism for emotional distress (Wang, 2022). For instance, a parent’s negative emotional response to a child’s frustration may increase their stimulation and teach them to suppress their feelings rather than understand and express them appropriately (Schultheis et al., 2019). Furthermore, abusive parents exhibit more negative emotions when interacting with their children and struggle to recognize and appropriately react to them (Wang, 2022). As a result, the infant has trouble recognizing their own emotions as well as those of others and develops maladaptive strategies to control undesirable feelings (Schultheis et al., 2019; Wang, 2022). Accordingly, VFO can interfere with secure attachment and reflective functioning’s regular development, promoting the use of pre-mentalistic modes of psychological functioning, such as excessive certainty, assumptions about mental states as if people are aware of other’s inner world (Condino et al., 2022); concrete thinking, failure to take into account complex mental states of oneself and others (Fonagy et al., 2016); teleological thought, the ability to identify mental states only when their effects are physically apparent (Fonagy et al., 2019); and intrusive pseudo-mentalization, a more hostile form in which feelings and thoughts are expressed with the intention to manipulate and control the lives of others (Condino et al., 2022; Garon-Bissonnette et al., 2023). Prementalistic ways of functioning hinder the social processes that facilitate human cooperation, such as creativity, negotiation, and consideration of others’ mental states. Indeed, to collaborate with others, it is necessary to put other people’s subjective states first, which limits the propensity to use violence to control other’s behaviors (Asen & Fonagy, 2017a).

Conversely, a deficit in reflective functioning seems to be linked with aggressive and violent behaviors (Abate et al., 2017; Parada-Fernández et al., 2023; Taubner et al., 2012), and with intimate partner violence more specifically (Mohaupt & Duckert, 2016; Stover et al., 2022). In these cases, violence may be seen as an immature and prementalistic strategy for the regulation of intense emotions and a means of shielding the victim from the fragmentation of self (Fonagy, 1999; Taubner et al., 2017). A similar function may be played by controlling behaviors as well. Due to reduced emotion regulation capacities, people with poor reflective functioning skills may have to expel intolerable mental states (such as negative emotions and cognitions) that threaten their integrity (Fonagy, 1999). The presence and proximity of the partner are required for such expulsion to occur, and the partner must be available to accept the expulsed mental states (Fonagy, 1999; Lorenzini et al., 2018). The proximity of the partner is necessary to regulate the affect that the individual is not able to regulate autonomously and is forced to expel to avoid psychic disorganization and restore autonomic functioning (Meloy, 1992). In this sense, controlling behaviors and especially isolating behaviors in people with low reflective capacity can serve to ensure the closeness of the partner and enable the expulsion of negative mental states in the absence of a more mature capacity for interpersonal distancing and emotion regulation. Furthermore, several studies emphasized the role of reflective functioning in the association between violence in the family of origin and violence in adolescence (Adler et al., 2020; Taubner et al., 2016; Taubner & Schröder, 2020) and IPV victimization in adulthood (Condino et al., 2022). Only Trombetta et al. (2024) specifically focused on the perpetration of intimate partner violence (i.e., physical, psychological, and sexual IPV), highlighting an indirect association between VFO and psychological IPV through reflective functioning. However, no studies have explored the role of reflective functioning in relation to the perpetration of isolating behavior.

Accordingly, the present study aims to assess the association between VFO and the perpetration of isolating behaviors, exploring the mediation role of reflective functioning. The data collected can provide clinical information to prevent and contrast the perpetration of isolating behaviors. As reflective functioning is described as a protective capacity that enables the processing of adverse circumstances, its development—for example, through mentalization-inspired treatment (see Asen & Fonagy, 2021)—can improve understanding of self and other mental states (Daubney & Bateman, 2015) and enable individuals to explore and integrate complex mental states and coherent emotion regulation strategies (Bateman & Fonagy, 2010; Daubney & Bateman, 2015). This, in turn, can reduce the perpetration of violent behaviors.

It should be noted that the data collected are part of an exploratory study and other variables (e.g., attachment styles, internal working models, and strategies of regulation of the attachment system; Velotti et al., 2018) may intervene in the relationship between VFO and IB perpetration. Therefore, in order to determine whether the two dimensions of reflective functioning—certainty and uncertainty of mentalization—may fully or partially mediate this relationship, the current study also examined the direct relationship between VFO and IB perpetration. In this regard, different theorethical approaches have pointed to the role of different mechanisms that can explain how exposure to IPV during childhood affects the likelihood of engaging in IPV in adulthood. Social learning theory, for example, emphasizes the role of the family context in reinforcing belief systems that legitimize the use of violence to resolve disputes and conflicts (Bandura, 1973), while Bowen’s family system theory highlights the lack of self-differentiation, inadequate family boundaries, and fusion in relationships as possible mechanisms of intergenerational transmission of violence (Bowen, 1978; Meyer et al., 2024). However, in line with a multidimensional approach to violence, such models are explanatory but not exhaustive in clarifying the relationship between VFO and the perpetration of IPV, and IB more specifically. Therefore, several risk factors at individual, relational, community, and sociocultural levels should be considered to fully explain the mechanisms involved in the intergenerational transmission of violence (Hardesty & Ogolsky, 2020).

### Hypotheses

**Figure 1:**
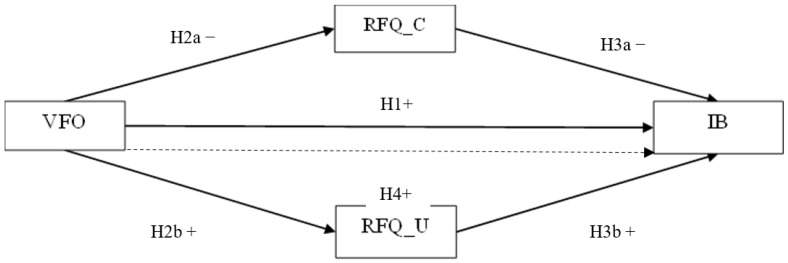
Hypothesized model. Note. VFO = Violence in the family of origin; RFQ_C = Reflective Functioning Questionnaire-certainty; RFQ_U = Reflective Functioning Questionnaire-uncertainty; IB = The perpetration of isolating behaviors. Positive and negative signs are indicative of the relationship between the variables. The solid arrow indicates the direct relationship between the study variables. The dashed arrow indicates the indirect relationship between VFO and IB mediated by RFQ_C and RFQ_U.

The current study was guided by four research hypotheses, also presented in Figure 1.

**H1:**  *Experienced violence in the family of origin should be directly and positively associated with the perpetration of isolating behaviors.*

**H2a:**  *Experienced violence in the family of origin should be directly and negatively associated with the certainty of mentalization (RF_C).*

**H2b:**  *Experienced violence in the family of origin should be directly and positively associated with the uncertainty of mentalization (RF_U).*

**H3a:**  *Certainty of mentalization should be directly and negatively associated with the perpetration of isolating behaviors.*

**H3b:**  *Uncertainty of mentalization should be directly and positively associated with the perpetration of isolating behaviors.*

**H4:**  *Uncertainty of mentalization and certainty of mentalization should mediate the association between violence in the family of origin and the perpetration of isolating behaviors.*

## 2. Materials and Methods

### 2.1. Procedure

The study’s methods follow the 1964 Declaration of Helsinki and the American Psychological Association’s ethical guidelines. The recruitment used snowball sampling. The questionnaire was administered online using LimeSurvey (version 3.27.22). To take part in the study, participants had to fulfill the following inclusion criteria: they had to be over 18 years old, understand Italian, and have been in a relationship within the last 12 months. Participation was entirely voluntary and anonymous. The questionnaire was disseminated by the research team members to their personal, professional, and social networks through email and word of mouth. An informed consent form outlining the study’s objectives and survey content, as well as the risks, benefits, privacy, names of research institutions, and contact details for the study team leader, was given to participants before the questionnaire started. The questionnaire took about 15 min to complete. The Bioethical Committee of the University of Turin gave its approval for the project (prot. n° 0429348, 9 July 2021).

### 2.2. Participants

The questionnaire was completed by 792 Italian participants. One hundred and twenty-nine participants were excluded because they had not been in a couple relationship in the last 12 months. The final sample included 663 Italian participants (68.5% female), aged between 18 and 76 (Mage = 28.38, *SD* = 8.51; 10 cases were missing for this variable), and involved in a couple relationship in the last 12 months. Table 1 presents the sample’s sociodemographic characteristics.

### 2.3. Measures

Violence in the family of origin (VFO): To measure experienced psychological and physical VFO, two items developed by the authors were used (a) “within your family of origin have you ever suffered threats, insults, or verbal offenses?”; (b) “within your family of origin have you ever suffered physical violence?—e.g., slaps, shoves, etc.”. Participants responded to each item on a 5-point Likert scale ranging from 1 (“never”) to 5 (“almost every day”). The scores for each item were summed to give a total score for experiences of VFO. According to Eisinga et al. (2013), since the measurement consists of only two items, the McDonald’s ω (or the Cronbach’s alpha) was not calculated as a measure of reliability (which tends to be an underestimated index in these cases), but rather the Spearman–Brown correlation. A strong correlation emerged between the two items (r: 0.61; *p* < 0.001).

Mentalization: To assess mentalization, the Italian version (Morandotti et al., 2018) of the Reflective Functioning Questionnaire (RFQ; Fonagy et al., 2016) was used. This instrument evaluates two dimensions of mentalization: certainty of mentalization (six items; RFQ_C), which assesses hypermentalization, and uncertainty of mentalization (six item; RFQ_U), which assesses hypomentalization. Low levels of certainty of mentalization correspond to maladaptive hypermentalizaing, while high levels refer to adaptive levels of certainty about mental states. On the contrary, low levels of uncertainty of mentalization correspond to adaptive opaqueness of mental states, while high levels refer to maladaptive hypomentalizing. In the questionnaire, participants were asked to read the statements presented carefully and mark the answer that best described them as a person for each one (items examples: “sometimes I do things without really knowing why” and “I don’t always know why I do what I do”, for RFQ_U and RFQ_C, respectively). Participants rated each item on a 7-point Likert scale ranging from 1 (“strongly disagree”) to 7 (“strongly agree”). For this study, the reliability was good (McDonald’s ω = 0.76 for RFQ_C and 0.97 for RFQ_U).

The perpetration of isolating behaviors: To assess the perpetration of isolating behaviors, the Revised Controlling Behavior Scale, and specifically the isolation subscale, was used (CBS-R; Graham-Kevan & Archer, 2005). This scale comprises 48 items (24 for perpetration and 24 for victimization) assessing controlling behaviors through five subscales: economic, threats, intimidation, emotional, and isolation. For the purposes of the present study, only the isolation perpetration subscale was used (six items; e.g., “I tried to reduce the time my partner spent with family or friends”). Participants were asked to indicate how often they or their partner had engaged in the reported behaviors in the past twelve months. The frequency of isolating behaviors was rated on a 5-point Likert scale that goes from 0 (“never”) to 4 (“very often”). For this study, the reliability was good (McDonald’s ω = 0.62).

### 2.4. Data Analysis

The R Statistical Software version 4.4.0 (R Core Team, 2024) was used to perform the statistical analyses, and a mediation model was tested using Hayes’ (2019) PROCESS macro (Version 4.1, Model 4). Frequencies, means, and standard deviations were computed to summarize the study variables. Pearson’s correlation (r) was used to test the relationship between variables, and Cohen’s (1988) conventions were used to interpret the results found. The reliability of each scale was determined using McDonald’s ω coefficient. The moderate amount of missing data in sociodemographic characteristics (e.g., education level) was handled by using a multiple imputation process with 10 iterations implemented through the Hmisc R package (Harrell, 2025).

The study variables were tested for the assumptions of normality and multicollinearity, as suggested by Tabachnick and Fidell (2007). Since the data violated the condition of multinormality, a robust estimator was used to test the significance of the model. To assess the significance of the indirect effects hypothesized in our mediation model, we used bootstrap estimation (Hayes, 2019) with 5000 samples, and we calculated the bias-corrected 95% confidence interval (CI) by determining the effects at the 2.5th and 97.5th percentiles. The indirect effects were significant when 0 was not included in the confidence interval. The bootstrap method has been shown to be a reliable test to identify indirect effects from a resampling process, especially with a small sample size (Hayes, 2019).

## 3. Results

Overall, 67.9% of participants had perpetrated at least one isolating behavior in the last 12 months. No significant gender differences emerged regarding the study variables (i.e., VFO, RF, IB). The bivariate correlations between the study variables as well as their mean values and standard deviations are reported in Table 2. All study variables were significantly correlated with each other, although the effect sizes of the identified correlations were small (except for the correlation between the two dimensions of the RFQ). Specifically, the results demonstrated that increases in levels of VFO were correlated with decreases in levels of certainty of mentalization, while increases in levels of VFO were correlated with increases in levels of uncertainty of mentalization and the perpetration of isolating behaviors. Furthermore, increases in levels of certainty of mentalization were correlated with decreases in levels of the perpetration of isolating behaviors, whereas increases in levels of uncertainty of mentalization were correlated with increases in levels of the perpetration of isolating behaviors. Finally, increases in levels of certainty of mentalization were correlated with decreases in levels of uncertainty of mentalization.

To test our hypotheses, a mediation model was tested with VFO as the independent variable, the two dimensions of the RFQ as mediators, and IB as the dependent variable. Age and education level were the only sociodemographic variables significantly and positively associated with the perpetration of isolating behaviors and were therfore included as covariates in the mediation model. All the hypotheses in the present study were confirmed. According to H1, higher levels of VFO predicted higher levels of IB (b: 0.22; SE: 0.06; *p* < 0.001). Furthermore, increased levels of VFO predicted decreased levels of RFQ_C, in line with H2a (b: −0.22; SE: 0.10; *p* < 0.05), and increased levels of RFQ_U (b: 0.15; SE: 0.06; *p* < 0.001), in line with H2b. H3a and H3b were confirmed as well: higher levels of RFQ_C predicted lower levels of IB (b: −0.14; SE: 0.02; *p* < 0.001), whereas higher levels of RFQ_U predicted higher levels of IB (b: 0.13; SE: 0.01; *p* < 0.01). According to H4, the indirect effects were also significant: increased levels of VFO indirectly predicted an increased level of IB through the mediation of RFQ_C (b: 0.011; Bootstrap SE: 0.015; 95% CI [0.001, 0.041]) and RFQ_U (b: 0.020; Bootstrap SE: 0.019; 95% CI [0.009, 0.067]). Finally, the total effect of VFO on IB was positive and significant (b: 0.32; SE: 0.06; *p* < 0.001). The model explained 9.7% of the variance for IB (F_5, 657_ = 13.643; *p* < 0.0001).

Table 3 synthesizes the mediation analyses’ results.

## 4. Discussion

The current study aimed to test the mediation role of the two dimensions of reflective functioning (i.e., certainty of mentalization and uncertainty of mentalization) in the association between violence in the family of origin (VFO) and the perpetration of isolating behaviors (IBs).

All hypotheses of the study were confirmed. According to H1, higher levels of VFO predicted higher levels of IB perpetration. Although little is known about how early exposure to violence can develop into a propensity for violence and especially isolating behaviors, previous research has established a link between violence in the family of origin and IPV perpetration (Eriksson & Mazerolle, 2014; Shakoor et al., 2022), in line with the results found in the present study. Indeed, childhood experiences of abuse and maltreatment consistently increased the risk of IPV perpetration in adulthood (Curtis et al., 2023; Haj-Yahia et al., 2021). According to attachment theory, adverse childhood experiences like violence in the family of origin can promote the development of insecure and disorganized attachment relationships and create internal working models of families that incorporate violence (Juan et al., 2020; McClellan & Killeen, 2000). As stated by Bowlby (1969/1982), infants who do not experience secure attachment may exhibit anger as a form of protest used to maintain or reestablish contact with the attachment figure. In adulthood, this type of behavior is often aimed at a romantic partner and can take the form of verbal abuse or coercive control (e.g., isolating behaviors) as a result of actual or perceived threats of rejection, separation, or abandonment from the partner (Bowlby, 1969/1982; Fonagy, 1999). However, it should be made clear that the intergenerational transmission of violence does not necessarily require direct experiences of abuse in the family of origin. In fact, several studies have shown that indirect experiences of violence (i.e., witnessing interparental violence) can also increase the likelihood of IPV perpetration in adulthood (Grest et al., 2018; Narayan et al., 2017). Although the aims of the present study did not include an assessment of witnessed violence, future research should include this form of violence in the family of origin to gain a broader understanding of the mechanisms regulating the intergenerational transmission of violence. According to H2a and H2b, increased levels of VFO predicted decreased levels of certainty of mentalization and increased levels of uncertainty of mentalization. Several studies (Asen & Fonagy, 2017a; Garon-Bissonnette et al., 2023; Stover et al., 2022) highlighted how negative childhood experiences, like violence in the family of origin, might affect reflective functioning skills and the related emotion regulation strategies. In abusive environments, caregivers may promote the development and adoption of harmful behaviors and maladaptive emotion regulation strategies in children rather than providing the support and scaffolding they need to learn healthy coping mechanisms and mature emotion regulation strategies (Kim & Cicchetti, 2010; Wang, 2022). In particular, VFO may disrupt the development of reflective functioning skills and encourage the use of pre-mentalistic modes of psychological functioning (i.e., excessive certainty, concrete thinking, teleological thought, and intrusive pseudo-mentalization; Condino et al., 2022; Garon-Bissonnette et al., 2023). These family contexts, which can be characterized by frightening or frightened caregivers, can encourage the child to internalize the caregiver’s feelings of fear, anger, or hatred, as well as their representation of themselves as terrifying and uncontrollable (Fonagy, 1999). Because they are too distressing, the child cannot think about these internalized representations and mental states. So, to avoid thinking, the child stops mentalizing, limiting their ability to regulate their emotion and to reflect on such negative mental states and representations (Asen & Fonagy, 2017a). However, while on the one hand the deactivation of mentalization in this frightening familiar context could be useful to protect the child from negative mental states that could endanger their integrity (Garon-Bissonnette et al., 2023), on the other hand this limits the ability to reflect on such negative mental states and representations, leading to deficits in reflective functioning skills (Asen & Fonagy, 2017a).

In turn, consistent with H3a and H3b, increases in the certainty of mentalization decreased the perpetration of isolating behaviors, whereas increases in the uncertainty of mentalization predicted an increase in the perpetration of isolating behaviors. Finally, considering H4, both dimensions of reflective functioning (i.e., certainty and uncertainty of mentalization) partially mediate the relationship between VFO and IB perpetration.

In adult intimate relationships, disrupted reflective functioning skills can lead to an intense need to control the partner and expel on him or her the negative mental states that the individual is not able to regulate autonomously with more mature mechanisms of emotion regulation (Fonagy, 1999). In this case, controlling the partner can allow for the expulsion of the intolerable parts of the self and the related negative mental states in order to maintain and preserve a cohesive and comfortable self-image (Fonagy, 1999; Lorenzini et al., 2018). Such externalization can work only if the individual is sufficiently able to perceive the intolerable parts of the self as external (Fonagy, 1999). One of the mechanisms through which this process can be made possible is, for example, projective identification, conceived as an immature defense mechanism also associated with deficits in reflective functioning (Bateman & Fonagy, 2004, 2006), useful for expelling negative and distressing parts of the self (Klein, 1946; Bion, 1962). In order for projective identification to take place and for the intolerable parts of the self to be expelled, the presence and closeness of the partner as an object of containment of these parts is necessary (Fonagy, 1999). Indeed, when the caregiver or the partner does not allow for the externalization of these intolerable parts, the individual can no longer defend themselves from psychic disorganization, resulting in a loss of the ability to think about others and themselves in a balanced manner (Asen & Fonagy, 2017a). Consequently, separation cannot be tolerated (Abrams, 2009; Willoughby, 2001). In this context, isolating behaviors can be seen as a dysfunctional and immature mechanism associated with poor reflective functioning, useful to avoid separation and the associated fear of abandonment (Palihawada et al., 2019) and to maintain closeness with the partner, which serves to ensure the expulsion of the intolerable parts of the self that threaten psychic disorganization (Fonagy, 1999).

However, it should be specified that only 9.7% of the variance in the perpetration of isolating behaviors was explained by the study variables, and reflective functioning only partially mediated the relationship between VFO and IB. This implies that the perpetration of isolating behaviors is influenced by additional variables and other mechanisms and factors can intervene. This is consistent with a multidimensional approach to violence that takes into account risk factors at several levels, including individual, relational, community, and sociocultural factors (Hardesty & Ogolsky, 2020).

### 4.1. Limitations

The study has several limitations. Firstly, the study’s cross-sectional design makes it difficult to explain the causality of the relationship between the variables. Furthermore, the assessment of RF focuses on current functioning, while the measurement of VFO and IB refers to prior experiences. Secondly, the study only looked at isolating behaviors, which limited the result’s applicability to other forms of IPV (physical, psychological, sexual IPV) and controlling behaviors, such as emotional and economic control and extreme jealousy and possessiveness. Thirdly, self-report questionnaires make the study susceptible to response biases. In addition, using an ad hoc questionnaire to measure VFO, which only assessed experiences of psychological and physical VFO (e.g., slaps, shoves, insults, and destroyed objects) and not other forms such as neglect, maltreatment, sexual abuse as well as witnessed violence limits the generalizability of the results found and a broader understanding of the mechanisms involved in the intergenerational transmission of violence. Fourthly, the higher proportion of female participants (68.5%) made the sample unbalanced in terms of gender distribution, and the use of a convenience sample of well-educated people with a good socioeconomic status could limit the generalizability of the present findings. Finally, the PROCESS macro for SPSS (Version 28) was used to test the direct and indirect effects in the present study. While this is appropriate for the study design (which included only manifest and no latent variables), it also has some limitations, mainly related to the lack of model fit indices.

### 4.2. Future Directions

Although the emerging results are encouraging, the current study has several limitations that could be taken into consideration for future research. Given the lack of literature on the role of mentalization in IPV perpetration, future studies should replicate the present study to support the preliminary findings that have further emerged. Furthermore, considering that reflective functioning partially mediates the association between VFO and IB perpetration, future research should explore other variables that may mediate this relationship (e.g., emotion regulation, attachment). For example, due to the strong theoretical and empirical link between reflective functioning and attachment, it could be relevant to include this variable in future studies as well. The inclusion of different forms of IPV and controlling behaviors (for example, including all the subscales of the CBS-R) could deepen the data found in relation to a broader range of aggressive and violent behaviors within the couple relationship. The inclusion of representative samples could increase the limited generalizability of the findings obtained in the present study. Similarly, future studies should include other forms of VFO such as witnessing violence, neglect, and sexual abuse. This could provide more insight into the mechanisms involved in the intergenerational transmission of violence and the role of different forms of violence in the family of origin. Furthermore, it may be useful to investigate gender differences and conduct separate analyses for each gender in future studies, also considering that women seem to show higher levels of mentalization compared to men (Poznyak et al., 2019; Schiffer et al., 2013; Tollenaar & Overgaauw, 2020) and that the relationship between violence in the family of origin and subsequent IPV perpetration seems stronger for men than for women (Smith-Marek et al., 2015). In addition, replication of this study in clinical samples could further support the usefulness of the findings found here in terms of intervention and treatment. Given the association between reflective functioning and mental health and personality disorders (e.g., borderline personality disorder), their inclusion in future analyses could deepen our understanding of the mechanisms involved in the intergenerational transmission of violence and IPV perpetration. Finally, the use of a longitudinal design could allow for confirmation of the causal directions hypothesized here, and the use of other statistical approaches that also provide model fit indices (e.g., AMOS) could further consolidate the results of the present study.

## 5. Conclusions

The preliminary findings of the present study contribute to advancing the understanding of the mediating role of mentalization in the relationship between violence in the family of origin and the perpetration of isolating behaviors. An indirect effect of VFO on the perpetration of IB through the certainty and uncertainty dimensions of mentalization emerged. The present findings support the hypotheses of the attachment theory and mentalization model on aggression and violence, extending them to the perpetration of isolating behaviors. Additionally, the preliminary findings that emerged can be relevant from both the clinical and research perspectives. As underlined by Dutton (2011), abusers’ problems with emotion regulation capacities and mentalization skills are rooted in early relationships, suggesting that the most significant and long-lasting consequences of family violence may not simply be the imitation of violent behaviors but rather the inability to regulate and control painful emotions. In this regard, interventions focused on the promotion of adequate reflective functioning skills, and mature emotion regulation strategies may help reduce controlling behaviors aimed at maintaining the closeness of the partner to regulate threatening psychological contents and defend the self from psychological disorganization. Specifically, mentalization-inspired approaches may help develop a more trusting attitude and lower levels of mindless violence by empowering the capacity to adjust to new ways of seeing things and to react to future changes (Asen & Fonagy, 2017b). The feeling that one’s perspective of reality is in line with another’s is what generally boosts confidence in the importance of practicing perspective-taking in general (Asen & Fonagy, 2021). Additionally, mentalization-inspired approaches can help to increase the ability to both experience and reflect on emotions, which is an essential component of emotion regulation (Weijers et al., 2016). Indeed, explicitly mentalizing an affective experience gives the individual the potential to express (or suppress) emotions in a non-automatic way (Weijers et al., 2016).

Although additional research is required to support the preliminary findings of the current study and the impact of experiences of violence in the family of origin on the perpetration of isolating behaviors, our results seem to suggest the usefulness of interventions informed by the mentalization model as well as the need for preventive measures against violence in the family of origin, in order to lower the risk of perpetrating intimate partner violence and isolating behaviors more specifically.

## Figures and Tables

**Table 1:** Characteristics of the sample.

		N	%
Sex			
	Female	454	68.5
	Male	209	31.5
Gender ^a^			
	Woman	443	66.8
	Man	203	30.6
	Transgender	8	1.2
	Other/Do not know	9	1.4
Sexual Orientation			
	Heterosexual	374	56.4
	Lesbian	59	8.9
	Gay	95	14.3
	Bisexual	66	10.0
	Prefer not to say	52	7.8
	Other	17	2.3
Educational level ^b^			
	Middle school diploma or less	10	1.6
	High school diploma	227	37.6
	Bachelor’s degree	259	42.9
	Master’s degree or higher	107	17.7
Employment status			
	Unemployed	23	3.5
	Freelancer	57	8.6
	Employee	180	27.1
	Student	397	59.9
	Homemaker	3	0.5
	Retired	3	0.5
Economic satisfaction ^c^			
	Insufficient	4	0.6
	Unstable	81	12.2
	Sufficient	327	49.4
	Wealthy or higher	203	37.7

Note N = 531. ^a^: 2 missing values. ^b^: 60 missing values. ^c^: 1 missing value.

**Table 2:** Mean scores, standard deviations, and bivariate correlations between the study variables.

	*M*	*SD*	1	2	3	4
1. VFO	5.30	2.47	—			
2. RFQ_C	6.82	4.95	−0.10 *	—		
3. RFQ_U	3.89	3.20	0.16 **	−0.45 **	—	
4. IB	8.49	2.99	0.18 **	−0.21 **	0.22 **	—

Note. N = 531. VFO = Violence in the family of origin; RFQ_C = Reflective Functioning Questionnaire-certainty; RFQ_U = Reflective Functioning Questionnaire-uncertainty; IB = The perpetration of isolating behaviors ** *p* < 0.001; * *p* < 0.05.

**Table 3:** Summary of direct, indirect, and total effects.

Path	β	B	(SE)	Bootstrap 95% CI
Direct Effects				
VFO → IB	0.143 ***	0.227 ***	0.062	[0.105, 0.349]
VFO → RFQ_C	−0.084 *	−0.222 *	0.105	[−0.429, −0.015]
VFO → RFQ_U	0.153 ***	0.261 ***	0.069	[0.124, 0.398]
RFQ_C → IB	−0.141 ***	−0.085 ***	0.021	[−0.128, −0.042]
RFQ_U → IB	0.135 *	0.126 *	0.010	[0.041, 0.211]
Indirect effects				
VFO → RFQ_C → IB	0.011 ^†^	0.019	0.015	[0.001, 0.041]
VFO → RFQ_U → IB	0.020 ^†^	0.033	0.019	[0.009, 0.067]
Total effect of VFO → IB	0.032 ***	0.279 ***	0.062	[0.157, 0.401]
Covariates				
Age ^a^ → IB	0.087 **	0.030	0.010	[0.004, 0.009]
Age ^a^ → RFQ_C	−0.032	−0.019	0.025	[−0.069, 0.031]
Age ^a^ → RFQ_U	0.035	0.013	0.013	[−0.012, 0.035]
Education ^b^ → IB	0.065	0.236	0.139	[−0.036, 0.065]
Education ^b^ → RFQ_C	0.115 **	0.696 **	0.229	[0.245, 0.115]
Education ^b^ → RFQ_U	−0.047	−0.183	0.156	[−0.491, 0.124]

Note: N = 663. SE = Standard Error; CI = Confidence interval; VFO = Violence in the family of origin; RFQ_C = Reflective Functioning Questionnaire-certainty; RFQ_U = Reflective Functioning Questionnaire-uncertainty; IB = The perpetration of isolating behaviors. *** *p* < 0.001; ** *p* < 0.01; * *p* < 0.05. ^†^: *p*-value not available for indirect effects. ^a^ = 10 imputed missing values. ^b^ = 60 imputed missing values.

## Data Availability

The dataset is available upon request to the authors.
